# Dental Consequences of Vitamin D Deficiency during Pregnancy and Early Infancy—An Observational Study

**DOI:** 10.3390/ijerph19041932

**Published:** 2022-02-09

**Authors:** Deanna M. Beckett, Jonathan M. Broadbent, Carolina Loch, Erin K. Mahoney, Bernadette K. Drummond, Benjamin J. Wheeler

**Affiliations:** 1 Department of Oral Sciences, Sir John Walsh Research Institute, University of Otago, Dunedin 9016, New Zealand; carolina.loch@otago.ac.nz; 2 Dental Department, Hutt Valley DHB, Lower Hutt 5040, New Zealand; erin.mahoney@huttvalleydhb.org.nz; 3 Department of Paediatrics and Child Health, University of Otago, Wellington 6242, New Zealand; 4 Department of Paediatric Dentistry, University of Leeds, Leeds LS2 9JT, UK; b.k.drummond@leeds.ac.uk; 5 Paediatric Endocrinology, Southern District Health Board, Dunedin 9016, New Zealand; ben.wheeler@otago.ac.nz; 6 Department of Women’s and Children’s Health, University of Otago, Dunedin 9016, New Zealand

**Keywords:** vitamin D, dental caries, developmental defects of dental enamel, 25-hydroxyvitamin D

## Abstract

Vitamin D (25OHD) status during pregnancy is closely correlated with foetal and new-born 25OHD. Calcification for primary teeth begins from the fourth month of intrauterine life and from birth for permanent teeth. Dental consequences of severe 25OHD deficiency are well documented; however, consequences are less documented for milder degrees of 25OHD deficiency. This study examined the dental consequences of vitamin D deficiency/insufficiency during gestation and infancy in a cohort of 81 New Zealand children. Pregnancy and birth data for the children and their mothers and 25OHD status during gestation, birth and at five months were obtained, and dental examinations were conducted. Associations between 25OHD and enamel defects or caries experience were investigated. Of the 81 children, 55% had experienced dental caries and 64% had at least one enamel defect present. Vitamin D insufficiency (25OHD < 50 nmol/L) at all timepoints was not associated with enamel defect prevalence, but during third trimester pregnancy it was associated with an increased caries risk IRR of 3.55 (CI 1.15–10.92) by age 6. In conclusion, maternal 25OHD insufficiency during the third trimester of pregnancy was associated with greater caries experience in primary dentition. No association was found between early life 25OHD and enamel defect prevalence or severity.

## 1. Introduction

Pregnancy is a unique and demanding time in terms of calcium and phosphate metabolism. For the developing foetus the third trimester is particularly important and is where much of the mineralisation of the foetal skeleton and the primary dentition occurs [1,2,3]. Vitamin D is one important part of this developmental process and plays a crucial role for mineral balance, with rapidly growing bone susceptible to mineralisation defects such as rickets [4]. To assist in maternal/foetal mineral and skeletal health, global recommendations are therefore to maintain maternal 25-hydroxyvitamin D (25OHD) levels during pregnancy above 30 ng/mL (74.9 nmol/L) [5,6]. However, despite these recommendations, vitamin D deficiency remains common during pregnancy [7,8]. This is important as infant vitamin D status at birth (and during gestation) is closely correlated with, and dependent upon, maternal status [7,9,10]. While the skeletal consequences of rickets and severe degrees of vitamin D deficiency in early childhood are well defined, the implications of lesser degrees of vitamin D deficiency/insufficiency, particularly during pregnancy and early neonatal life, are less well understood.

Dental health may offer some insights into these questions. Dental enamel is formed by ameloblasts and is the most mineralised tissue in the human body, with calcium and phosphate identified as important for enamel mineralisation during early tooth development and also for remineralisation of erupted teeth [11]. As with bone, the dental manifestations arising from severe vitamin D deficiency have been well described, with defects including enamel and dentine mineralisation abnormalities, large pulp chambers with high pulp horns and periapical abscesses unrelated to dental caries or dental trauma [12,13,14,15]. A 2013 systematic review examined the link between childhood vitamin D supplementation and dental caries and identified that supplemental vitamin D could be associated with up to 47% reduced risk of dental caries, although inconsistencies between various studies were apparent [16]. The influence of vitamin D with developmental enamel defects has also been studied, which has produced inconsistent and contradictory findings [17,18,19].

Given the current lack of strong clinical evidence for negative skeletal and dental health outcomes arising from mild to moderate vitamin D deficiency during pregnancy and early life, this study aims to examine the potential dental consequences (both caries and mineralisation defects) of varying degrees of early life vitamin D deficiency, using maternal third trimester and infant 25OHD levels.

## 2. Materials and Methods

One-hundred and twenty 5-to-6-year-old children who participated in an earlier 2012 NZ observational study [8,16] and randomized controlled trial (RCT) [6] examining maternal and infant vitamin D status following postnatal maternal vitamin D supplementation during breastfeeding, were invited to participate in this study. For this dental health study, inclusion criteria were participation in the previous 2012 study and being at least 5.5 years of age at time of dental assessment. Exclusion criteria were inability to cope with a comprehensive dental examination.

A full description of the methods of the previous studies is available [6,8]; but in summary, inclusion criteria were pregnant women and their babies (post birth); and exclusion criteria were premature delivery (prior to 37 weeks gestation), intent to use postnatal vitamin D supplementation, and a history of disorders known to affect calcium and/or vitamin D metabolism. While these participants were taken from an RCT, no study intervention occurred prior to the postnatal age of 1 month, and no intervention to the infants was undertaken.

For the current dental health study, parental informed consent and child assent were obtained, and a comprehensive dental examination was provided by a registered dental professional (DB) at the Faculty of Dentistry, University of Otago. Calibration of dental assessments was provided by a second researcher (EM). Clinical dental assessments were performed blinded to prior vitamin D status. Comprehensive pregnancy and birth data for mothers and babies, maternal 25OHD status during the third trimester of pregnancy and infant status from birth (cord blood) and at five months, were sourced from the 2012 studies [6,8]. Baseline demographic and medical information available through the 2012 RCT were updated, and oral health information were collected via survey to determine dental caries and enamel defect risk, including history of trauma, exposure to sources of fluoride, diet and homecare habits. Deprivation was measured using NZ Deprivation index 2018 (NZDep2018) [20]. Diet data included the number of daily servings of sweet foods and drinks that were reported by participants for the previous month.

Primary and permanent teeth were assessed wet and dry for observation of dental caries and enamel defects. Digital posterior bitewing radiographs were taken unless available within in the previous twelve months from the child’s usual dental provider. Dental caries in the enamel and dentine were classified using standardised World Health Organization (WHO) criteria and the International Caries Detection and Assessment System (ICDAS-II) [21,22]. The decayed, missing and filled teeth (dmft) index was used to document dental caries [23]. Primary dmft and permanent DMFT mean scores were combined to present a complete mixed dentition count which is reported as dmft/DMFT. Many children lose their primary incisors at age six; therefore, when primary teeth only are reported, canines, first and second molars were included in the count (12 teeth in total) as these were the primary teeth participants had in common. Radiographically evident enamel and dentine caries were coded using the caries classification system ICDAS-II. Radiographs were read by two calibrated paediatric dentists (BD, EM), and a third clinician (DB) mediated if required.

Enamel defects were classified using both the European Academy of Paediatric Dentistry guidelines for diagnosis of MIH (EAPD for MIH) and the Modified Defect of Dental Enamel Index (DDE Index) [24,25]. Training and calibration for EAPD for MIH and DDE was provided by a paediatric dentist (EM), and a recheck of 20% of randomly selected participants was conducted by EM to ensure consistency. Enamel defects greater than 1mm on the buccal, occlusal or lingual surface of all primary and permanent teeth present were recorded, including demarcated opacities, diffuse opacities and hypoplasia. Atypical restorations or extractions due to an enamel defect were also recorded.

Most data are presented as means, standard deviations and percentages. The count of dmft/DMFT caries-affected teeth is a continuous variable, often used to characterize the severity of dental decay. This variable was re-categorized into caries free (0 caries-affected teeth), low caries experience (1–2 affected teeth) and high caries experience (3+ caries-affected teeth). Enamel defect severity was characterized by the count of teeth affected by developmental enamel defects. Total 25OHD levels (D2 + D3 combined) from maternal third trimester, birth (cord blood), and 5-month infant bloods were re-categorized as: Sufficiency ≥ 50 nmol/L; Insufficiency < 50 nmol/L; and Deficiency ≥ 30 nmol/L. Associations between categorical 25OHD were investigated in association with: (1) developmental enamel defects; (2) caries experience. Associations between independent variables were tested for statistical significance using the chi-square test for categorical variables while non-parametric analyses (such as the Kruskal-Wallis H test or Mann-Whitney U test) were used for continuous dmft/DMFT caries severity counts that were positively skewed. Associations between cord, pregnancy and 5-month 25OHD that had been dichotomized into insufficient (score 1) and sufficient (score 0), and continuous dental measures (including dmft indices and DDE index), were investigated using negative binomial regression (NBR), and Incidence Rate Ratios (IRR) with confidence intervals were calculated. To manage one dmft/DMFT score outlier, a sensitivity analysis was conducted whereby the highest dmft/DMFT score was recoded to the next highest dmft/DMFT score. Bivariable was investigated as well as multivariable models adjusting for sex, birthweight and season of delivery.

Statistical analyses were conducted using Stata 16.0 (StataCorp, College Station, TX, USA) and two-sided *p* < 0.05 was considered statistically significant.

## 3. Results

Full participant and maternal demographics are presented in Table 1. At the time of dental assessment, the mean age for participants was 6.6 years (SD ± 0.6). Just under half of all participants lived in areas of medium deprivation, with those living in areas of high and low deprivation well represented at 20 and 37%, respectively. Seasons of birth were well represented, although slightly more babies were born in winter (33%), and fewer were born in summer (16%). All infants were born at term.

Participant 25OHD data across three time periods are presented in Table 2. 25OHD data were available for most children at birth and at 5 months-of-age; however, maternal bloods were not available for 24 participants (30%) during the third trimester of pregnancy. The greatest level of 25OHD deficiency was observed at 5 months-of-age, with five participants having levels below 10 nmol/L, the lowest being 4.7 nmol/L. At birth, the lowest level observed was 13.9 nmol/L, and during the final trimester of pregnancy, the lowest maternal level was 26.6 nmol/L.

Just over half of all participants (55%) had experienced dental caries, with nearly a third (28%) classified as having high caries experience (dmft 3+) in the primary dentition (Table 3). Caries severity (dmft/DMFT scores) was higher for participants whose mothers had insufficient 25OHD levels in the 3rd trimester, compared to those with sufficient levels, at 3.3 (SD 5.0) and 1.5 (SD 1.8) respectively, although this difference was not statistically significant.

Participants with insufficient 25OHD in cord blood also had higher mean dmft/DMFT scores when compared to those who were sufficient in 25OHD, although this difference was less than for maternal blood. No difference in dmft/DMFT scores was observed when looking at insufficient and sufficient 25OHD levels at 5-months-of-age. Approximately two thirds of participants had at least one enamel defect present, with demarcated opacities being the most observed defect type (58%). The mean number of demarcated opacities in participants who had at least one opacity observed was 4.8 (SD 3.3).

Children whose mothers had third trimester 25OHD insufficiency (<50 nmol/L) had 3.6 times the rate (IRR) of dental caries at age six than those whose mothers had sufficient 25OHD (≥50 nmol/L). This finding held after controlling for sex, birthweight and season of delivery (Table 4). Sugar intake was not associated with either outcome and was not included in the model. After recoding the highest dmft/DMFT score (17) to the next highest dmft/DMFT score (9) as a sensitivity analysis, the dental caries rate (IRR) for children whose mothers had insufficient 25OHD in their third trimester reduced slightly to 3.0, which was still statistically significant (*p* < 0.05). There was no change to IRR for clinically apparent enamel defects of any type when looking at 25OHD levels at any time.

## 4. Discussion

This study identified that participants with mothers who had 25OHD insufficiency during the third trimester of pregnancy had over three times the rate (IRR 3.55) of dental caries at age 6, compared to children whose mothers had 25OHD sufficiency in the third trimester of pregnancy. Cord blood and 5-month serum 25OHD levels were not significant predictors for dental caries. No associations were observed between 25OHD levels at any time point and the development of any type of dental enamel defect (IRR 0.37–0.69).

Possible implications of milder degrees of vitamin D insufficiency/deficiency are important for ongoing discussions around importance for pregnancy and infant vitamin D supplementation and provide some additional support in favour of supplementation, particularly highlighting further evidence in support of potentially substantially reduced caries experience. They also highlight the value of dental health as a potential marker of overall mineral and skeletal health in early life. Many studies have investigated vitamin D in association with enamel defects and/or dental caries experience, and in the absence of 25OHD information, methods often include vitamin D supplementation or self-reported vitamin D intake from dietary sources [16,18,26]. A systematic review investigating vitamin D and dental caries in controlled clinical trials identified that supplemental vitamin D before the age of 13 has been associated with a reduced risk of caries by up to 47. Although, the inability to identify a dose-response relationship and the lack of available serum vitamin D levels were identified as important weaknesses [16].

Recently, there have been three dental health studies published (one only as a conference proceeding) with access to prenatal and early life 25OHD data through previous longitudinal studies [17,27,28]. Suarez-Calleja et al. obtained 25OHD from the INMA-Asturias birth cohort to assess dental caries prevalence in 188 children aged between 6 and 10 [27]. Maternal 25OHD were available from 12 weeks gestation and child 25OHD at 4 and 8 years-of-age. The study found that 25OHD insufficiency (<50 nmol/L) in the first trimester of pregnancy and in children at 8 years-of-age were risk factors for dental caries, with odds ratios of 2.51 and 3.45, respectively [27]. Navarro et al. (2021) [28] utilised 25OHD data from a cohort study in The Netherlands (*n* = 5257), with maternal serum levels available from the second trimester and from participants at birth and at age 6. Participants with severe prenatal and early childhood serum 25OHD deficiencies (<25 nmol) were more likely to experience dental caries than those with mild or no deficiency, but this association was weak [28]. Both of these studies utilised prenatal 25OHD prior to the third trimester, whereas our study utilised third trimester 25OHD, as this is when the majority of primary tooth enamel mineralisation occurs [3]. All three studies identified an association between prenatal 25OHD and dental caries prevalence by age 6, although the strength of the associations differed, which could be due to the timing of 25OHD collection. Like our study, Naverro et al. also investigated 25OHD at birth and found no association with dental caries experience at age 6. A Norwegian longitudinal study by Borsting et al. (2019) investigated enamel hypomineralisation in 855 children aged 7–9 years, using available maternal serum 25OHD obtained during the second and third trimester of pregnancy as a marker for vitamin D levels [17]. Similar to our findings, no statistically significant associations between enamel hypomineralisation/dental enamel defects of any type and maternal 25OHD were identified at maternal second or third semester serum levels nor cord blood or with cut-offs of 50 nmol/L and 75 nmol/L.

### 4.1. Strengths and Weaknesses

The main strength of this study is the availability of serum 25OHD levels at three critical time points during dental development. Calcification of the primary teeth begins from the fourth month of intrauterine life and at birth for the first permanent molar, central incisors and lower lateral incisors, making the third trimester and cord blood an opportune time for accessing mineralisation and formation defects that may develop [3]. A limitation of the study was the restricted number of participants, which was a pre-determined number based on available 25OHD data from the original study [8]. Missing 25OHD data for some timepoints was another limitation; however, data were available for most participants (70% at 3rd trimester, 96% at birth and 91% at 5 months), and those with missing data from one time point were still able to be included in the analysis. The participants were also largely from a homogeneous European ethnicity.

The distribution of participants identified as having sufficient, insufficient and deficient levels of 25OHD was both a strength and weakness. At birth, an even distribution between the three cut points was observed, meaning that clear comparisons between groups could be made. However, in the third trimester, 25OHD had to be dichotomised into insufficient (<50 nmol/L) and sufficient (≥50 nmol/L) to allow for appropriate analyses. Nevertheless, this fulfilled the primary aims of investigating the impacts of milder degrees of vitamin D deficiency on early life. Finally, due to the varied age of eruption of permanent teeth for participants in this study, many did not have all their permanent incisors and first molars. This meant that Molar Incisor Hypomineralisation (MIH) could not be assessed in relation to vitamin D status [24]. Enamel defects in general were still able to be assessed, however, and all teeth present in the mouth (primary and permanent) were included in the analysis.

### 4.2. Implications and Future Recommendations

Teeth affected by enamel defects are more susceptible to post eruptive breakdown and dental caries [11,29]. Those with untreated dental caries can be subject to judgment and blame for having the disease, with modifiable factors such as poor diet and oral hygiene often cited as the reason for their suffering [30]. Identifying factors that may affect enamel development can enable researchers to identify supplemental interventions during pregnancy that could positively impact dental development for their unborn babies, reducing the risk of dental caries or tooth breakdown after tooth eruption.

Understanding the relationship between 25OHD and early life dental health can contribute to discussions on the value of vitamin D supplementation during pregnancy and early life. There is increasing data supporting supplementation with evidence now spanning prevention of nutritional rickets and respiratory diseases [9,31,32,33]. This data continues to build the dental evidence of benefit, with this data and others suggesting potential benefits in preventing dental caries +/− developmental enamel defects [16,18]. Given the considerable detrimental health burden poor dental health has for later adult life this data has importance beyond teeth alone [29,30].

Debate around the impacts of milder degrees of deficiency/insufficiency for subsequent child and adult health are ongoing, and dental health research may be able to contribute to knowledge in this area to provide further supporting or refuting evidence of the impacts of milder degrees of deficiency [6].

This study recommends that existing studies with late pregnancy and cord blood 25OHD consider dental assessments to provide increased data for meta-analysis in the future. Ideally, we would also do further dental follow-up between 7 and 8 years-of-age, to enable permanent incisors and first molars to be assessed for enamel defects in conjunction with primary canines, first and second molars. This would allow for complete MIH screening data.

## 5. Conclusions

This study provides new data suggesting maternal vitamin D insufficiency during the third trimester of pregnancy is associated with a considerably greater caries experience in primary dentition by age 6 years. However, no association was found between early life 25OHD and enamel defect prevalence or severity. This data further strengthens recommendations for pregnancy and early life vitamin D supplementation and supports and builds on the known bone and mineral benefits of healthy vitamin D status.

## Figures and Tables

**Table 1:** Characteristics of participating children and their mothers by sex. N (%).

Characteristics	Male	Female	All
**Participant Characteristics**			
Deprivation Index ^a^			
Low	17 (40)	13 (33)	30 (37)
Med	15 (36)	20 (51)	35 (43)
High	10 (24)	6 (15)	16 (20)
Ethnicity			
NZ European	40 (95)	31 (79)	71 (88)
NZ Māori	0 (0)	6 (15)	6 (7)
Other	2 (5)	2 (5)	4 (5)
Mean gestation in weeks (SD)	39.7 (1.2)	39.8 (1.0)	39.7 (1.1)
Season of Birth			
Spring	11 (26)	10 (26)	21 (26)
Summer	5 (12)	8 (21)	13 (16)
Autumn	13 (31)	7 (18)	20 (25)
Winter	13 (31)	14 (36)	27 (33)
Birthweight (gms)			
2600–2999	3 (7)	6 (15)	9 (11)
3000–3999	30 (71)	28 (72)	58 (72)
4000–4900	9 (21)	5 (13)	14 (17)
Mean Birthweight (gms)	3591 (503)	3534 (461)	3564 (481)
**Maternal Characteristics**			
Age at delivery			
17–24 years	2 (5)	2 (5)	4 (5)
25 to 34 years	23 (55)	22 (56)	45 (56)
35+ years	17 (40)	15 (38)	32 (40)
Mean age at delivery	32.9 (4.9)	34.0 (4.1)	33.4 (4.6)
Total	42 (52)	39 (48)	81 (100)

^a^ Deprivation Index was defined according NZDep Index 2018.

**Table 2:** Participants’ 25OHD levels: deficiency, insufficiency and sufficiency.

25OHD ^a^	N (%)	nmol/L Range
Third Trimester (maternal blood)		
Deficient	3 (4)	26.6 to 29.6
Insufficient	11 (14)	30.8 to 47.7
Sufficient	43 (53)	51.5 to 160
Total ^b^	57 (71)	26.6 to 160
Birth (child cord blood)		
Deficient	28 (35)	13.9 to 29.1
Insufficient	25 (31)	30.4 to 49.3
Sufficient	25 (31)	50.0 to 98.8
Total ^c^	78 (97)	13.9 to 98.8
5 months (child blood)		
Deficient	16 (20)	4.7 to 29.5
Insufficient	11 (14)	30.6 to 44.7
Sufficient	47 (58)	51.6 to 156
Total ^d^	74 (92)	4.7 to 156

^a^ 25OHD nmol/L: Deficient > 30, Insufficient 30–49, Sufficient 50+. ^b^ Missing data for 24 participants (30%), mean 250HD total sample = 72.3 nmol/L (SD 30.6). ^c^ Missing data for 3 participants (4%), mean 25OHD total sample = 41.6 nmol/L (SD 20.4). ^d^ Missing data for 7 participants (9%), mean 25OHD total sample = 71 nmol/L (SD 41.0).

**Table 3:** Vitamin D data by dental caries and enamel defects.

Characteristics	3rd Trimester—Maternal Blood			Birth—Cord Blood			5 Months—Infant Blood			Total
	Deficient <30 nmol	Insufficient ^a^ <50 nmol	Sufficient 50+ nmol	Deficient <30 nmol	Insufficient ^a^ <50 nmol	Sufficient 50+ nmol	Deficient <30 nmol	Insufficient ^a^ <50 nmol	Sufficient 50+ nmol	
All	3 (5)	14 (17)	43 (53)	28 (36)	53 (65)	25 (31)	16 (22)	27 (33)	47 (58)	81 (100)
Caries experience ^b^										
Caries free	0 (0)	5 (35)	19 (44)	12 (35)	24 (67)	10 (40)	5 (15)	12 (44)	22 (46)	36 (44)
Low	1 (5)	5 (35)	14 (33)	5 (24)	13 (25)	8 (32)	7 (33)	10 (37)	11 (23)	22 (27)
High	2 (14)	4 (29)	10 (23)	11 (48)	16 (30)	7 (28)	4 (21)	5 (19)	14 (30)	23 (28)
Enamel defect prevalence ^c^										
Any defect	2 (5)	8 (15)	30 (58)	15 (30)	31 (60)	19 (36)	11 (23)	17 (33)	30 (58)	52 (64)
Any demarcated opacity	2 (6)	7 (15)	27 (57)	14 (31)	28 (60)	17 (36)	9 (21)	14 (30)	28 (60)	47 (58)
Demarcated W/C	2 (6)	7 (16)	26 (58)	13 (30)	26 (58)	17 (38)	9 (23)	14 (31)	26 (58)	45 (56)
Demarcated Y/B	1 (6)	3 (13)	13 (57)	8 (35)	15 (65)	8 (34)	4 (18)	5 (22)	17 (74)	23 (28)
Diffuse	0 (0)	1 (6)	11 (69)	3 (20)	6 (38)	9 (56)	1 (7)	3 (19)	11 (69)	16 (20)
Caries severity ^d^										
dmft/DMFT	9.3 (7.5)	3.3 (5.0)	1.5 (1.8)	2.8 (4.0)	2.2 (3.2)	1.6 (1.7)	1.9 (1.9)	1.4 (1.8)	2.1 (3.3)	1.9 (2.8)
dmft for common teeth ^e^	6.7 (6.1)	2.6 (4.0)	1.4 (1.8)	2.5 (2.4)	1.9 (2.8)	1.6 (1.7)	1.7 (2.0)	1.2 (1.8)	1.9 (2.8)	1.8 (2.5)
Enamel defect severity ^f^										
Any defects	3.0 (2.8)	4.4 (2.6)	7.3 (4.8)	5.7 (4.2)	5.9 (4.6)	6.6 (4.2)	6.3 (3.9)	4.9 (3.7)	7.1 (4.6)	6.1 (4.3)
Any demarcated opacity	3.0 (2.8)	4.0 (2.2)	5.3 (3.6)	4.2 (2.3)	4.8 (3.5)	4.8 (3.2)	5.7 (2.5)	4.3 (2.8)	5.5 (3.5)	4.8 (3.3)
Demarcated W/C	1.5 (0.7)	2.7 (2.1)	3.2 (1.8)	2.8 (1.7)	3.0 (1.9)	3.2 (1.7)	4.0 (1.9)	3.1 (1.8)	3.3 (1.8)	3.1 (1.8)
Demarcated Y/B	3.0 (0.0)	3.0 (2.0)	3.1 (2.2)	2.5 (1.4)	2.5 (1.3)	2.8 (2.9)	3.0 (1.8)	2.6 (1.8)	2.7 (2.1)	2.6 (1.9)
Diffuse	0.0 (0.0)	4.0 (0.0)	3.3 (2.8)	5.0 (3.6)	3.3 (2.9)	3.4 (2.2)	4.0 (0.0)	2.7 (1.2)	3.1 (2.5)	3.3 (2.4)

dmft = count of decayed, missing and filled teeth. dmft denotes primary dentition. (DMFT) denotes permanent dentition. (dmft/DMFT) denotes mixed dentition. ^a^ Insufficient includes deficient in this table—all participants with 25OHD < 50 nmol. ^b^ Caries experience = n (%) determined by dmft score. Caries free = score 0. Low = score 1–2. High = score 3+. ^c^ Reported as n (%). ^d^ Reported as mean (SD). ^e^ Common teeth include primary canines, first and second molars only (total of 12 teeth included in calculation). These are teeth that children aged 6 all have regardless of dental age of permanent tooth eruption. ^f^ Mean no. enamel defects among those with defects. W/C = white or cream coloured. Y/B = yellow or brown coloured.

**Table 4:** Incidence Rate Ratio (IRR) with confidence intervals (CI) for dental caries and enamel defects by vitamin D insufficiency (<50 nmol/L).

	IRR (CI) Maternal Vitamin D: Third Trimester	IRR (CI) Baby: Cord Blood	IRR (CI) Baby: 5 Months	IRR (CI) Ever Insufficient ^c^	IRR (CI) Ever Deficient ^d^
dmftDMFT ^a^					
Model 1	2.31 (1.00–5.34) ^e^	1.42 (0.69–2.93)	0.68 (0.32–1.43)	1.19 (0.51–2.79)	1.65 (0.86–3.15)
Model 2	2.35 (1.01–5.44) ^e^	1.42 (0.69–2.93)	0.68 (0.32–1.45)	1.18 (0.50–2.78)	1.63 (0.85–3.13)
Model 3	3.55 (1.15–10.92) ^e^	1.67 (0.68–4.06)	0.90 (0.36–2.26)	1.22 (0.48–3.10)	1.64 (0.81–3.31)
Enamel defects ^b^					
Model 1	0.46 (0.19–1.11)	0.65 (0.32–1.29)	0.69 (0.35–1.36)	0.63 (0.29–1.39)	0.71 (0.38–1.33)
Model 2	0.46 (0.18–1.16)	0.67 (0.33–1.36)	0.65 (0.33–1.30)	0.65 (0.30–1.41)	0.70 (0.37–1.30)
Model 3	0.37 (0.13–1.06)	0.55 (0.21–1.47)	0.67 (0.27–1.63)	0.61 (0.24–1.57)	0.75 (0.39–1.43)
Demarcated opacities					
Model 1	0.54 (0.22–1.36)	0.67 (0.32–1.39)	0.67 (0.33–1.39)	0.61 (0.27–1.37)	0.65 (0.34–1.24)
Model 2	0.54 (0.21–1.40)	0.69 (0.33–1.44)	0.65 (0.32–1.35)	0.61 (0.27–1.39)	0.64 (0.34–1.24)
Model 3	0.47 (0.16–1.36)	0.67 (0.24–1.86)	0.62 (0.24–1.58)	0.63 (0.23–1.73)	0.70 (0.36–1.38)

Model 1 = controlling for sex. Model 2 = controlling for sex and birthweight. Model 3 = controlling for sex, birthweight and season of birth. ^a^ dmft/DMFT = mean number of decayed filled or missing teeth in primary and permanent teeth (full mouth count). ^b^ Includes all enamel defect types. ^c^ If participants have ever had a 25OHD of <50 nmol/L at any of the measured times. ^d^ If participants have ever had a 25OHD of <30 nmol/L at any of the measured times. ^e^ *p* < 0.05—negative binomial regression.

## Data Availability

The data that support the findings of this study are available from the corresponding author, D.M.B., upon reasonable request.
